# Complete mitochondrial genome of the skinnycheek lantern fish *Benthosema pterotum* (Perciformes: Myctophidae) in the East China Sea

**DOI:** 10.1080/23802359.2019.1698980

**Published:** 2019-12-13

**Authors:** Liang Zheng, Yayuan Xiao, Yafei Duan, Min Yang, Xia Yang, Yongmin Mu, Wenquan Sheng, Yanming Sui

**Affiliations:** a Chinese Academy of Fishery Sciences East China Sea Fishery Research Institute, Shanghai, China;; b South China Sea Fisheries Research Institute, Guangzhou, China;; c Shanghai Ocean University, Shanghai, China;; d People’s Hospital of Linyi, Linyi, China;; e Shanghai Municipal Public Security Bureau, China

**Keywords:** *Benthosema pterotum*, mitochondrial genomem, Myctophidae

## Abstract

The complete mitochondrial genome sequence of *Benthosema pterotum* is first described in this article. The total length of mitogenome is 18,052 bp. It contains 13 protein-coding genes, 22 tRNA genes, and two ribosomal RNA genes. The overall base composition of H-strand is 27.83% A, 30.88% C, 25.61% T, and 15.69% G, with an A＋T bias of 53.43%. The phylogenetic analysis result showed that the *B. pterotum* and *Electrona carlsbergi* were close relationship.

The skinnycheek lantern fish *Benthosema pterotum* (Alcock), of the family Myctophidae, is widely distributed in the subtropical-tropical waters including East China Sea (Sassa et al. 2010). They are small in size ranging from 2 to 30 mm (Homaei et al. 2013). *Benthosema pterotum* feed on a variety of zooplankton, including copepods as the most important food item (Dalpadado and Gjøsaeter 1988; Valinassab et al. 2007).

The complete mitochondrial genome of *B. pterotum* first determined in this paper was expected to provide help on population genetics of *B. pterotum* and further molecular phylogenetic studies. The sample of *B. pterotum* in this article was collected from the East China Sea (121°56′E, 30°52′N) and stored in the East Sea Fisheries Research Institute Fish Specimen Room (Accession number: MBp201904100172). Its DNA was frozen at −80 °C in the Key Laboratory of East China Sea Fishery Resources Exploitation, Ministry of Agriculture, China. According to genes from *B. pterotum*, 12S ribosomal RNA gene (Accession: LC146182), 16S ribosomal RNA gene (Accession: KR231720), cytochrome c oxidase subunit I (COI) gene (Accession: JX133773), cytochrome b (Cytb) gene (Accession: JX133771) primers were designed, and PCR amplification and sequencing were conducted.

The whole length of *B. pterotum* mitogenome was 18,052 bp and submitted in GenBank (Accession No. MN266306). The nucleotide composition of the heavy strand was 27.83% for A, 30.88% for C, 25.61% for T, and 15.69% for G, with a high A＋T bias of 53.43%. It contains 13 protein-coding genes, 22 tRNAs, and two rRNAs. Most genes were located on the heavy strand, but *ND6* and 8 tRNA genes (*tRNA^Gln^*, *RNA^Ala^*, *tRNA^Asn^*, *tRNA^Tyr^*, *tRNA^Cys^*, *tRNA^Ser^*, *tRNA^Glu^*, *tRNA^Pro^*) were encoded on the light strand. Most protein-coding genes initiated with ATG except for COI starting with GTG. Four types of protein-coding genes ended with typical termination codons TAA (ND1, ATPase 8, ATPase 6, COIII, ND4L), TAG (ND3, ND5, ND6), AGA (COI), and T–– (ND2, COII, ND4, Cytb). The length of 12S (located between *tRNA^Glu^* and *tRNA^Met^*) and 16S (located between *tRNA^Phe^* and *tRNA^Pro^*) rRNA genes were 952 bp and 1683 bp, respectively.

To investigate the phylogenetic relationship, we downloaded the mitochondrial genome sequences of 17 currently available species. The concatenated sequences of 13 protein-coding genes, two rRNAs genes, and 22 tRNAs genes were aligned with the ClustalW program (Larkin et al. 2007). Using the Maximum-Likelihood (ML) method (Stamatakis 2006), the phylogenetic tree was constructed (Figure 1) using MEGA6 (Tamura et al. 2013). The best-fitting model (GTR + I + G) was obtained as the optimization model using jModelTest (Posada 2008). The result indicating that the *B. pterotum* and *Electrona carlsbergi* were close relationship (Figure 1).

**Figure 1.**
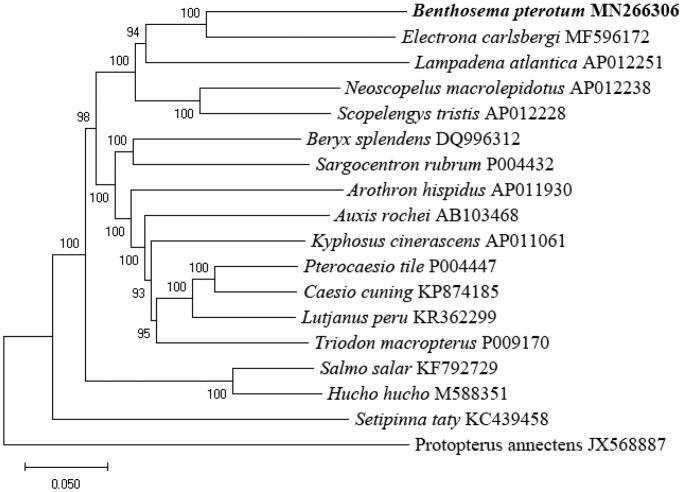
The phylogenetic tree based on the 13 protein-coding genes, two rRNAs genes, and 22 tRNAs genes of *Arothron hispidus, Auxis rochei, Beryx splendens, Caesio cuning, Electrona carlsbergi, Hucho hucho, Kyphosus cinerascens, Lampadena atlantica, Lutjanus peru, Neoscopelus macrolepidotus, Pterocaesio tile, Salmo salar, Sargocentron rubrum, Scopelengys tristis, Setipinna taty, Triodon macropterus*, and an outgroup *Protopterus annectens*. The bootstrap supports for Maximum-Likelihood (ML) method was indicated at each branch.
